# Publisher Correction: Cdh2 coordinates Myosin-II dependent internalisation of the zebrafish neural plate

**DOI:** 10.1038/s41598-019-43789-0

**Published:** 2019-05-17

**Authors:** Claudio Araya, Hanna-Maria Häkkinen, Luis Carcamo, Mauricio Cerda, Thierry Savy, Christopher Rookyard, Nadine Peyriéras, Jonathan D. W. Clarke

**Affiliations:** 1 Laboratory of Developmental Biology, Instituto de Ciencias Marinas y Limnológicas, Facultad de Ciencias, Universidad Austral de Chile, Campus Isla Teja, 5090000 Valdivia, Chile; 2 Centro Interdisciplinario de Estudios del Sistema Nervioso (CISNe), UACh, Valdivia, Chile; 3 Anatomy and Developmental Biology Program, Institute of Biomedical Sciences, Faculty of Medicine, Universidad de Chile, PO Box 70031, Santiago, Chile; 4 Biomedical Neuroscience Institute, Independencia, 1027 Santiago, Chile; 5 UPS3611 Complex Systems Institute Paris Ile-de-France (ISC-PIF), CNRS, Paris, France; 6 USR3695 BioEmergences, CNRS, Paris-Saclay University, Gif-sur-Yvette, France; 7 Centre for Developmental Neurobiology, Institute of Psychiatry Psychology and Neuroscience, King’s College London, New Hunt’s House, Guy’s Campus, London, SE1 1UL United Kingdom

Correction to: *Scientific Reports* 10.1038/s41598-018-38455-w, published online 12 February 2019

The original PDF version of this Article contained truncated versions of Figures 4 and 7. These errors have now been corrected in the PDF version of the Article; the HTML version was correct from the time of publication.

