# Supplementary material for: Cdh2 coordinates Myosin-II dependent internalisation of the zebrafish neural plate
Source: Sci Rep. 2019 Feb 12;9:1835. doi: 10.1038/s41598-018-38455-w (PMC6372647; doi:10.1038/s41598-018-38455-w)
Supplement: Supplementary file 1 — Supplementary Material [file 41598_2018_38455_MOESM1_ESM.pdf]

Cdh2 coordinates Myosin-II dependent internalisation of the zebrafish neural plate

Claudio Araya, Hanna-Maria Häkkinen, Luis Carcamo, Mauricio Cerda, Thierry Savy, Christopher Rookyard, Nadine Peyriéras, and Jonathan D W Clarke.

Supplementary Figure Legends

Figure S1. Depletion of *cdh2* leads to defective neurulation and abnormal apico-basal polarity

(**a**-**b**) Maximum projections of dorsal confocal pictures showing abnormal neural tube morphology and disorganised apical midline seam specialisation in *cdh2/pac* by 24 hpf, as revealed by apical marker, ZO-1 (arrows). Anterior is up. (**c**-**d**) Cross-section analysis in *cdh2/pac* mutants reveal disrupted apical midline seam organisation (arrowheads) at hindbrain levels.

Figure S2. Cdh2 turnover through zebrafish neurulation

(**a**) Dorsal single confocal picture of zebrafish neural plate at 9 hpf showing Cdh2-fast/dynamic "green" turnover in TgBAC(*cdh2*:*cdh2*-tFT)[24]. (**a'**) Dorsal single confocal picture of zebrafish neural plate at 9 hpf showing n-cadherin slow/stable "red" turnover in TgBAC(*cdh2*:*cdh2*-tFT). (**b**) Dorsal confocal picture of zebrafish neural plate at 12 hpf showing Cdh2 "green" turnover. (**b'**) Dorsal confocal picture of zebrafish neural plate at 12 hpf showing Cdh2 "red" turnover. (**c**) Dorsal confocal picture of zebrafish neural tube at 18 hpf showing Cdh2 "green" turnover. (**c'**) Dorsal confocal picture of zebrafish neural tube at 18 hpf showing Cdh2 "red" turnover. Arrowhead indicates tissue midline.

Figure S1. Depletion of *cdh2* leads to defective neurulation and abnormal apico-basal polarity

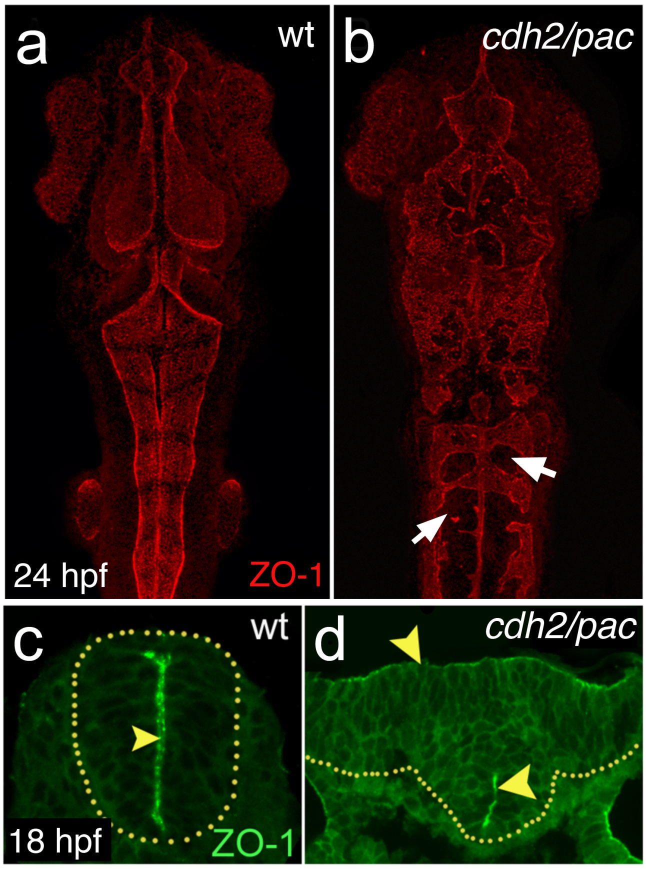

Figure S2. Cdh2 turnover through zebrafish neurulation

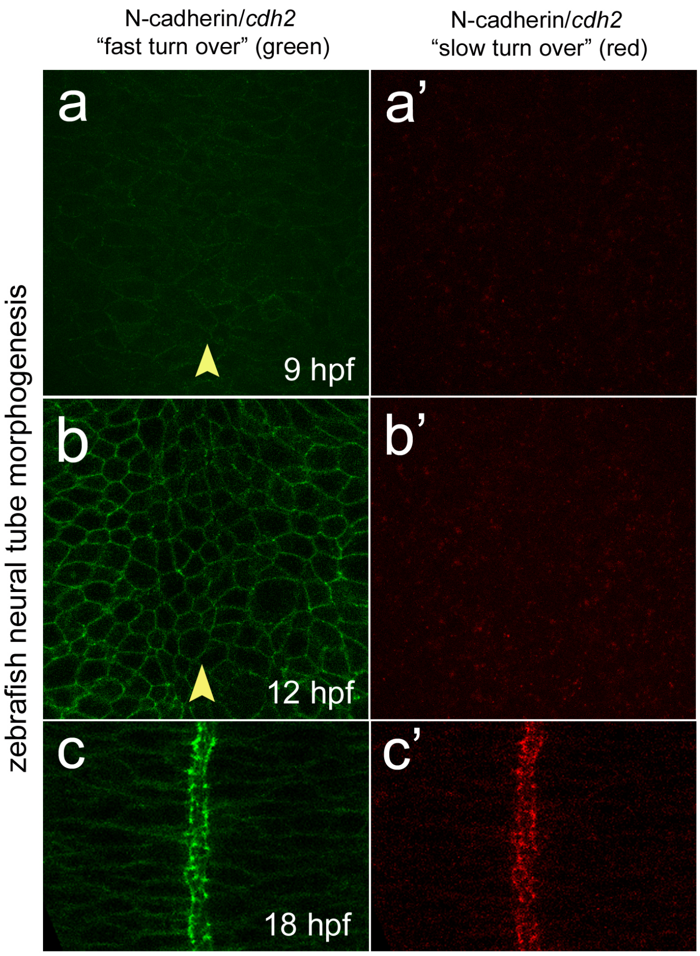

Movie Legends

Movie 1. Tissue morphogenesis during zebrafish hindbrain neurulation. Cells expressing GFP-caax to label membranes. Movie collected from 10-17.5 hpf and covers neural plate convergence and internalisation, transition to neural keel and then to neural rod. Arrowhead indicates midline and ov indicates otic vesicles as anatomical landmark for hindbrain. Dorsal to top. Frames are every 5 minutes. Time is in hours and minutes from start of movie.

Movie 2. Nuclei tracking reconstructed from embryo expressing H2B-GFP. during zebrafish neural plate internalisation. Dorsal view movie of a wild-type embryo from approximately 10-14 hpf covers convergence and internalisation of neural plate. Anterior to top. Arrowhead indicates midline. Frames are every 2.5 minutes.

Movie 3. Dorsal cell surface dynamics in medial zone of neural plate. Segmented representation of the dorsal surface profile of medial neural plate cells derived from membrane labelled cells, individual profiles cannot always be followed from frame to frame. Movie from 11-12 hpf covering period of cell internalisation in medial zone. Colour coded so larger cell surfaces are light grey and smaller surfaces are darker greys. Arrowhead indicates midline. Frames are every 5 minutes.

Movie 4. Dorsal cell surface dynamics in lateral zone of neural plate. Segmented representation of the dorsal surface profile of lateral neural plate cells derived from membrane labelled cells, individual profiles cannot always be followed from frame to

frame. Movie from 11-12 hpf. Colour coded so larger cell surfaces are light grey and smaller surfaces are darker greys. Arrowhead indicates direction of movement towards medial zone. Frames are every 5 minutes.

Movie 5. Myosin-II localisation during plate to keel transition. Single transverse view timelapse movie of a transgenic Tg(actb1:myl12.1-GFP) zebrafish embryo. Yellow arrows indicate high levels of Myosin-GFP at the dorsal surface of medial zone during period of internalisation. Arrowhead indicates midline. Frames are every 5 minutes.

Movie 6. Tissue morphogenesis during neural plate morphogenesis in cdh2/pac mutant embryo. Transverse view time-lapse movie from 10-15.15 hpf. Notice that during neural keel stages tissue fails to internalise and the neural anlage assumes a "T-shape" configuration. Arrowhead indicates midline and ov indicates otic vesicles as anatomical landmark for hindbrain. Frames are every 5 minutes. Time is in hours and minutes from start of movie.

Movie 7. Abnormal Myosin-II localisation during neural plate morphogenesis in Cdh2 deficient embryo. Transverse view time-lapse movie of a transgenic Tg(actb1:myl12.1-GFP) zebrafish embryo injected with cdh2 morpholino. Yellow arrows indicate high levels of Myosin-GFP across a wide expanse of dorsal surface of neural plate. Arrowhead indicates midline. Frames are every 5 minutes.

Movie 8. Dorsal cell surface dynamics across neural plate of cdh2/pac mutant embryo. Segmented representation of the dorsal surface profile of neural plate cells

derived from membrane labelled cells, individual profiles cannot always be followed from frame to frame. Movie from 11.5-12.5 hpf. Colour coded so larger cell surfaces are light grey and smaller surfaces are darker greys. In contrast to wt embryos, small cell surface area profiles are more evenly spread across mediolateral axis. Arrowhead indicates midline. Frames are every 5 minutes.
